# The Discovery and Characterization of Targeted Perikaryal-Specific Brain Lesions With Excitotoxins

**DOI:** 10.3389/fnins.2020.00927

**Published:** 2020-09-08

**Authors:** Joseph T. Coyle, Robert Schwarcz

**Affiliations:** ^1^McLean Hospital, Harvard Medical School, Belmont, MA, United States; ^2^Maryland Psychiatric Research Center, University of Maryland School of Medicine, Baltimore, MD, United States

**Keywords:** α-amino-3-hydroxy-5-methyl-4-isoxazolepropionic acid receptor, excitotoxins, glutamic acid, ibotenic acid, kainic acid receptor, N-Methyl-D-aspartate receptor, muscimol, quisqualic acid

## Abstract

The neurotoxic action of glutamic acid was first described by Lucas and Newhouse, who demonstrated neural degeneration in the inner layers of the neonatal mouse retina after systemic treatment with L-glutamate. Olney extended these findings by showing that neuronal degeneration affected other brain structures including neurons within the arcuate nucleus of the hypothalamus and the area postrema, that the lesion spared axons passing through these areas, and that the neurotoxic potency of glutamate analogs correlated with their excitatory potency, resulting in the designation “excitotoxins.” As this method affected only a small number of brain regions, it was not suitable for targeted brain lesions. The Coyle laboratory showed that direct injection of the potent glutamate receptor agonist, kainic acid, into the rat striatum caused a rapid degeneration of intrinsic neurons while sparing axons of passage or termination including the unmyelinated dopaminergic terminals. Kainic acid also exhibited this perikaryal-specific and axon-sparing profile when injected into the cerebellum, hippocampus and eye. However, neuronal vulnerability was highly variable, with hippocampal CA3, pyriform cortex and amygdala neurons exhibiting great sensitivity due to kainate’s high convulsive activity. In a comparison study, ibotenic acid, a potent glutamatergic agonist isolated from the *amanita muscaria* mushroom, was found to have excitotoxic potency comparable to kainate but was far less epileptogenic. Ibotenate produced spherical, perikaryal-specific lesions regardless of the site of injection, and experiments with specific glutamate receptor antagonists showed that its effects were mediated by the N-methyl-D-aspartate receptor. Because of this uniform neurotoxicity and near ubiquitous efficacy, ibotenic acid became the excitotoxic lesioning agent of choice. The discovery of the excitotoxic properties of the tryptophan metabolite quinolinic acid and of the anti-excitotoxic, neuroprotective effects of the related metabolite kynurenic acid in the Schwarcz laboratory then gave rise to the concept that these endogenous compounds may play causative roles in the neuropathology of a wide range of neurological and psychiatric disorders.

## Introduction

Lesioning neurons of interest has long been a tool in neuroscience research to define their physiologic and behavioral roles as well as their synaptic connections. These lesions may be accomplished by electrolytic (thermal) probes or transection of axon pathways. Degenerating axons become argentophilic, permitting the tracing of their pathways in brain sections to their fields of synaptic termination (Fink and Heimer, 1967). Brain lesions can be used to ascribe particular functions after the loss of the neurons targeted by the lesion (Fritz et al., 2016). However, interpreting the consequences of these destructive lesions is complicated by the fact that the lesion will damage axons passing through or terminating in the lesion site, causing antero- and retrograde degeneration. The concern remains whether the changes observed post-lesion result from the loss of the neurons of interest or are the unintended consequences of damage to other neuronal systems, perhaps distant from the lesion. Thus, a method of lesioning that could be precisely targeted to kill only the neurons of interest would greatly facilitate identifying the role of specific neurons.

At the time, another challenge in neuroscience research was to recreate in experimental animals the neuropathology characteristic of neurodegenerative disorders. These disorders typically involve a very selective degeneration of a population of neurons while sparing of adjacent neurons and axons of passage – unlike penetrating lesions or strokes. For example, Parkinson’s disease affects the dopaminergic neurons in the substantia nigra (Hornykiewicz, 1973); Huntington’s disease primarily affects the striatal GABAergic neurons (Perry et al., 1973; Bird and Iversen, 1974); and amyotrophic lateral sclerosis targets the cholinergic motor neurons (Rowland, 1991). Thus, our understanding of Parkinson’s disease was greatly facilitated by the development of 6-hydroxydopamine (6-HODA), which is specifically concentrated into catecholaminergic neurons. Mediated by their pre-synaptic high affinity transporters (DAT in dopaminergic neurons and NET in noradrenergic neurons; Uretsky and Iversen, 1969; Zis et al., 1974), 6-HODA accumulates in the catecholaminergic neurons, causing toxic oxidation and covalent binding to intracellular proteins (Saner and Thoenen, 1971). However, such specificity to neurons based on their neurotransmitter identity has been quite limited to catecholaminergic (6-HODA), serotonergic (5,7-dihydroxytryptamine; Gately et al., 1986) and cholinergic neurons (AF64A; Sandberg et al., 1984).

In this chapter, we describe the development of stereotaxic injection of potent and specific ionotropic glutamate receptor agonists to cause perikaryal-specific, axon-sparing lesions to define the roles of specific neuronal systems and to model neurodegenerative disorders. The lesion method, originally based on the potent glutamate receptor agonist kainic acid, was refined with the introduction of ibotenic acid, which provides a more uniform perikaryal-specific lesion. The *in situ* excitotoxin lesion method has been widely used in basic and translational neuroscience research for over 40 years (Table 1).

**TABLE 1:** Seminal publications on excitotoxin-induced brain lesions.

Lucas and Newhouse (1957), this study first described the selective degeneration of retinal neurons after systemic treatment with monosodium glutamate. Cited > 830 times.
Olney (1969a), this study demonstrated neuronal degeneration in the hypothalamus resulting from neonatal treatment of mice with monosodium glutamate that resulted in a striking phenotype including stunted growth, blindness and obesity. Cited >1,400 times.
Olney et al. (1971), this study demonstrated a close correlation between excitatory potency and neurotoxicity of systemically administered analogs of L-glutamate, prompting the description of this form of neuronal degeneration as “excitotoxicity.” Cited >760 times.
Coyle and Schwarcz (1976), this study first demonstrated that direct injection of a potent excitatory analogs of L-glutamate, kainic acid, into the brain parenchyma caused degeneration of surrounding neuronal cell bodies but spared axons of passage and of termination, causing a pattern of neuronal degeneration in the striatum resembling the pathology of Huntington disease. Cited >1,090 times.
Coyle et al. (1978), this study more fully documented the perikaryal-specificity of the kainate lesion using light, histofluorescence, electron microscopy and neurochemical analyses. Cited > 490 times.
Campochiaro and Coyle (1978), this study indicated that the vulnerability of striatal neurons to kainate coincided with the development of [3H]kainate receptor binding and Na^+^-dependent [^3^H]-L-glutamate transport, a marker for glutamatergic innervation. Cited >240 times.
Biziere and Coyle (1978), as evidence suggested that kainate receptors may have a presynaptic localization on glutamatergic terminals, this study demonstrated that prior removal of cortical glutamatergic projections to the striatum prevented kainate-induced neuronal toxicity. Cited >490 times.
Nadler et al. (1978), this study found a tremendous variation in neuronal vulnerability to kainic acid with hippocampal pyramidal neurons showing great sensitivity. Cited >650 times.
Schwarcz et al. (1979), this study found that ibotenic acid, with excitatory potency similar to kainic acid, caused a uniform, spherical perikaryal-specific lesion without seizures or distant neuronal degeneration. Cited >400 times.
Schwarcz et al. (1983), this study revealed that quinolinic acid, a metabolite of L-tryptophan that is an NMDA receptor agonist, produced perikaryal-specific neuronal degeneration after intra-striatal injection. Cited >1,110 times.
Foster et al. (1984), this study was the first to show anti-excitotoxic (= neuroprotective) properties of an endogenous glutamate receptor antagonist, and suggested that the close metabolic relationship between kynurenic acid and quinolinic acid may be of relevance for the pathophysiology of human neurodegenerative disorders. Cited >400 times.
Choi (1992), this paper provides a comprehensive review of the molecular mechanisms mediating excitotoxicity. Cited >1,900 times.

## Excitotoxicity of Systemic Glutamate Treatment

The excitotoxin lesion technique derived from the observations originally made over 60 years ago by Lucas and Newhouse (1957) on the cytotoxic effects of systemically administered glutamic acid on the retina of the neonatal mouse. The experiments were motivated by the belief that L-glutamate might have therapeutic effects in preventing hereditary degeneration of the retina. Contrary to expectations, the investigators observed degeneration of the neurons in the inner layer of the retina in the mice treated with systemic glutamate. This retinal inner layer consists primarily of horizontal, bipolar, and amacrine cells, which are interneurons. Subsequently, Olney described the acute degeneration not only of the retinal interneurons (Olney, 1969b) but also of neurons with cell bodies located in the arcuate nucleus of the hypothalamus and in the circumventricular organs after monosodium glutamate (MSG) was systemically administered to the neonatal rodent (Olney, 1969a). Of note, the arcuate nucleus and circumventricular organs lie outside the blood-brain barrier.

Olney carried out electron microscopic studies of the evolution of the MSG lesion in the mouse retina and arcuate nucleus and observed that massive swelling of the neuronal dendrites and perikaryal represented initial neuropathologic alterations (Olney, 1971). Notably, axons passing through the arcuate or even terminating on affected neurons remained impervious to MSG. Around the same time, glutamate and structurally related acidic amino acids were shown to have potent excitatory effects (Curtis and Watkins, 1963; Johnston et al., 1974) and to cause influx of sodium and water as well as efflux of potassium in cerebral cortical slices incubated *in vitro* (Harvey and McIlwain, 1968). In a structure-activity relation study in the arcuate nucleus of the neonatal mouse, Olney et al. (1971) demonstrated a correlation between neuroexcitatory potency and neurotoxic effects of a large series of systemically administered analogs of glutamate. He concluded that the neurotoxic effects of these glutamate analogs result from their depolarizing action, which caused influx of sodium and water into dendrites bearing “glutamate receptors.” Accordingly, he coined the term “excitotoxins” for these substances that appeared to kill neurons on the basis of their excitatory effects.

## Beyond a Generic Ionotropic Glutamate Receptor

In order to understand the basis for developing the *in situ* excitotoxin lesion technique, it is essential to appreciate the knowledge base of glutamatergic function nearly 50 years ago. Beginning in the 1950s, neurophysiologists demonstrated that iontophoretically applied glutamic acid and related acidic amino acids excited virtually all neurons within the central nervous system (CNS) (Hayashi, 1954; Curtis et al., 1959; Curtis and Watkins, 1963). These amino acids depolarized neurons by activating specific sites concentrated on dendrites and perikarya (Szentágothai, 1971). The discovery of a specific, high-affinity and sodium-dependent uptake process for glutamate on synaptosomes (Balcar and Johnston, 1972; Bennett et al., 1972) led to the suggestion that glutamate may serve as an excitatory neurotransmitter within the mammalian CNS. Neurophysiologists, initially working on the invertebrate neuromuscular junction (Gerschenfeld, 1973), which is glutamatergic, and then in the mammalian CNS characterized the electrophysiologic behavior of a number of natural products and known toxins that resembled glutamate (Johnston et al., 1974).

In a review of these studies, Biscoe et al. (1976) noted that excitatory moieties invariably contain two separate acidic groups, which lose protons and become negatively charged at physiologic pH, and at least one basic group, which becomes positively charged at physiologic pH. In all the moieties tested, one of the acidic groups was a carboxylic acid, whereas the other group was capable of considerable variation, including carboxyl, sulfonic, sulfinic, phosphonic, hydroxamic or other groups attached to an electron-withdrawing system. The acidic groups were separated by the length equivalent to two-three carbons. The basic substituent was invariably an α-amino group situated next to an acidic residue.

The potent excitatory effects of conformationally restricted and synthetic analogs such as kainic acid, quisqualic acid, ibotenic acid and N-methyl-D-aspartic acid (NMDA) led to the proposal that more than one receptor might mediate the action of these glutamate analogs (Johnston et al., 1974; McCulloch et al., 1974). Precise definition of a receptor was facilitated by specific antagonists, which at the time were mostly derivatives and analogs of glutamate. Using iontophoretic and bath application of antagonists, McLennan and his colleagues (Haldeman and McLennan, 1972; McLennan and Lodge, 1979) and Watkins and his colleagues (Davies et al., 1979) identified three glutamate receptor subtypes. One, which is activated by NMDA, is specifically antagonized by D-α-aminoadipate (Hicks et al., 1978) and by Mg^2 +^ (Nowak et al., 1984). Another receptor responds to quisqualate and is relatively insensitive to inhibition by D-α-aminoadipate but is antagonized by glutamate diethyl ester. Finally, excitation produced by the iontophoretic application of kainic acid was antagonized neither by D-α-aminoadipate nor by glutamate diethyl ester. Ibotenate’s effect was most consistent with being a NMDA receptor agonist. This proposal of three ionotropic glutamate receptors proved prescient as cloning studies many years later confirmed three molecularly distinct families of glutamate receptors: the NMDA receptor, the kainate receptor and the α-amino-3-hydroxy-5-methyl-4-isoxazolepropionic acid (AMPA) (formerly named quisqualate) receptor (Seeburg, 1996; Mayer, 2017).

## *In situ* Lesioning With Kainic Acid

The first *in situ* injection of an excitotoxin to produce a perikaryal-specific lesion was motivated by an interest in reproducing the pathology of Huntington’s disease (HD). The prominent symptoms of HD, a choreo-athetotic movement disorder complicated by progressive dementia, may result from degeneration of neurons primarily in basal ganglia (Bruyn, 1968). In the 1960s, several research groups applied post-mortem neurochemical analyses, focusing on biochemical markers associated with specific neurotransmitter-associated entities that had been so informative with regard to understanding the dopaminergic deficits in Parkinson’s disease (Hornykiewicz, 1973). By the mid-1970s, these studies had identified several neurochemical abnormalities in HD associated with specific neurotransmitter systems. Perry et al. (1973) reported marked reductions in the levels of GABA in the caudate, globus pallidus, and substantia nigra in HD. Bird and Iversen (1974) confirmed the GABAergic deficits in the caudate-putamen but also revealed cholinergic deficits, reporting significantly reduced activity of choline acetyltransferase (ChAT), the synthetic enzyme for acetylcholine. In contrast, they found that the concentration of dopamine and the specific activity of its synthetic enzyme, tyrosine hydroxylase (TH), were unaffected. Thus, consistent with the histopathology, HD appeared to affect the intrinsic GABAergic and cholinergic neurons while sparing the myelinated axons traversing the caudate-putamen and the dopaminergic terminals projecting from the substantia nigra.

Based on the insights from these post-mortem studies, one of us (JTC) hypothesized that the apparent “axon-sparing” striatal lesions in HD may be excitotoxic in nature. Direct injection of a potent glutamate receptor agonist could therefore induce perikaryal-specific neurodegeneration similar to HD in experimental animals. To test this idea, we chose kainic acid, reputed to be among the most potent glutamate receptor agonists (Buu et al., 1976), as an experimental tool. Isolated from the seaweed *digenia simplex*, kainic acid was used medically as an ascaricide and was known as a potent agonist at the crayfish neuromuscular junction (Shinozaki and Shibuya, 1974). An initial study of the effects of an intrastriatal injection of kainic acid, using doses from 0.5 to 5.0 μg in the adult rat, showed dose-related reductions of ChAT activity, a marker for cholinergic neurons, and glutamate decarboxylase (GAD) activity, a marker for GABAergic neurons, 48 h after injection; in contrast, TH activity, used as a marker for striatal dopaminergic terminals, exhibited a dose-dependent acute *increase* in the same tissues (Coyle and Schwarcz, 1976; Schwarcz and Coyle, 1977b). In controls, injection of the non-specific toxin, copper sulfate, caused a parallel decline in all three markers. Neurochemical analyses of the striatum 10 days after the injection of 2.5 μg of kainic acid confirmed the striking decline in cholinergic and GABAergic markers and the sparing of extrinsic dopaminergic afferents. Microscopically, cresyl violet-stained sections from the lesioned striatum revealed massive loss of striatal intrinsic neurons accompanied by a proliferation of reactive astrocytes whereas the myelinated axon bundles traversing the striatum were spared (Coyle and Schwarcz, 1976).

A detailed histopathologic study of the effects of an intra-striatal kainate injection revealed a spherical lesion that evolved over time (Coyle et al., 1978). During the first 48 h, intrinsic neurons exhibited chromatolysis, clumping of nuclear chromatin followed by disruption of the nuclear membrane. Beginning 1 week after injection, there was a marked proliferation of reactive astrocytes in the gray matter previously occupied with intrinsic neurons. Histofluorescence microscopy revealed that the dense dopaminergic terminal field in the striatal lesion did not degenerate. Electron microscopic analysis indicated that the corticofugal myelinated axons traversing the lesioned striatum, too, were unaffected. Many presynaptic boutons, presumably of extrinsic origin, were intact up to 10 days after injection; and osmophilic vestiges of post-synaptic elements remained adherent to these boutons. Thus, confirming the prior neurochemical studies, these results provided direct evidence of the perikaryal-specificity of the *in situ* kainic acid lesion, which offered “a new technique for making selective brain lesions that will be useful for examining neuronal connectivity” (Coyle et al., 1978). Follow-up studies confirmed the perikaryal-selective lesions with *in situ* kainic acid injections in cerebellum (Herndon and Coyle, 1977), retina (Schwarcz and Coyle, 1977a) and hippocampus (Schwarcz et al., 1978b).

## Mechanism of Excitotoxic Lesion

Although Olney had demonstrated a reasonable correlation between excitatory potency and neurotoxicity in the arcuate nucleus among a variety of glutamate analogs injected systemically, variables such as absorption, metabolism and brain access complicated interpretation (Olney et al., 1971). Schwarcz et al. (1978a) therefore compared the neurotoxic potency of several glutamate analogs after *in situ* injection in the striatum in the rat and intra-ocular injection in the chick. They also observed a rough correlation between excitatory potency and *in vivo* neurotoxicity (kainate > ibotenate > quisqualate > NMDA > > > glutamate). Glutamate, which is ~50-fold less potent than kainate in electrophysiologic studies *in vitro* (Shinozaki and Shibuya, 1974), was in fact virtually non-toxic at a dose 1700-fold higher than kainate – likely due to its rapid removal by cellular uptake (Bennett et al., 1972). Consistent with prior electrophysiologic findings (Biscoe et al., 1976), the kainate derivatives dihydrokainate, N-acetyl-kainate and kainate diethylester, which lack excitatory potency, were devoid of neurotoxic effects.

Subtle changes in kainate’s stereoisomerism, orientation of its side chain or saturation of the double bond on the side-chain dramatically changed its electrophysiologic properties. Thus, kainic acid appeared to act with high affinity at a very selective receptor (Shinozaki and Shibuya, 1976). Using [3H]-kainic acid with high specific radioactivity, London and Coyle (1979) characterized a high and a low affinity specific binding site for [3H]-kainic acid on rat brain membranes that correlated with the relative affinities of kainate analogs in electrophysiologic studies. This strongly supported the inference that these sites represented functional kainate-sensitive subtypes of ionotropic glutamate receptors. Prior kainic acid lesion of the striatum reduced the number of these sites by > 60 percent, indicating that the majority of kainate receptors reside on neurons intrinsic to the striatum. The regional distribution of kainate receptors was quite uneven (striatum > frontal cortex = hippocampus > cerebellum > pons-medulla). Notably, > 90 percent of the specific binding in the cerebellum and the pons-medulla was to the low affinity site.

To further characterize the mechanism of kainate toxicity, Campochiaro and Coyle (1978) examined the age-dependency of the vulnerability of striatal neurons to an *in situ* injection of kainate in rats. At 7 days post-partum, minimal degeneration of intrinsic cholinergic and GABAergic neurons was observed. By postnatal day 10, modest excitotoxicity occurred. Vulnerability increased substantially during the second and third postnatal week but was still less pronounced than in adulthood. Correspondingly, specific binding of [3H]-kainate was only 6% of adult levels at 7 days after birth. During the subsequent 2 weeks of maturation, there was a dramatic 10-fold increase in receptor binding, with the number of sites increasing from 1.6 to 21.6 fmol/mg of tissue between 1 and 3 weeks post-partum. By 4 weeks, receptor density reached 80% of adult levels. Together with the fact that the receptor resides on vulnerable neurons, this developmental pattern provided strong evidence for its critical role in kainate-induced striatal excitotoxicity.

The presence of the kainate receptor was obviously necessary for kainate toxicity to occur *in vivo*. But was it sufficient? We noted that the ontogenetic development of vulnerability of striatal neurons to kainate paralleled the development of cortico-striatal glutamatergic fibers (Campochiaro and Coyle, 1978), and that the neuronal susceptibility to kainate in the cerebellum tracked closely with glutamatergic innervation (Herndon and Coyle, 1977). Moreover, studies in invertebrates had indicated presynaptic effects of kainate on glutamatergic afferents (Shinozaki and Shibuya, 1974). We therefore decided to explore the possibility that glutamatergic afferents to the striatum play a role in kainate neurotoxicity. Indeed, we found that decortication, which removes the major glutamatergic innervation of the striatum (Divac et al., 1977), prevented the neurotoxicity normally caused by an intra-striatal injection of 10 nmol of kainic acid in adult rats (Biziere and Coyle, 1978). Although 1 μmol of glutamate was devoid of excitotoxicity when injected into the striatum on its own, its co-injection with 10 nmol of kainate restored kainate’s neurotoxicity in the denervated striatum (Biziere and Coyle, 1979). Notably, decortication also prevented the behavioral response to a unilateral intra-striatal kainate injection (rotation), and this effect, too, was restored by co-injecting glutamate. McGeer et al. (1978) independently replicated these findings of the neuroprotective effect of decortication but ascribed it to the fact that kainate was a glutamate uptake inhibitor. This inference was refuted by showing that dihydrokainate, a much more potent glutamate transport inhibitor, did not restore kainate excitotoxicity in the denervated striatum (Biziere and Coyle, 1978).

## Limitations of the *in situ* Kainate Lesion Method

The decortication experiments demonstrated that kainate’s excitotoxicity is profoundly affected by glutamate. Because kainic acid causes seizures following systemic administration in mice (Olney et al., 1974), and because of the emerging evidence that increased glutamate may be causally involved in seizure phenomena (Bradford and Dodd, 1977), it became apparent that systemic, intraventricular or local injection of kainate may cause a pattern of nerve cell loss similar to that seen in the brain of epilepsy patients (Corsellis, 1970). All these treatments indeed resulted in neurodegeneration in the hippocampus and pyriform cortex closely resembling the histopathology of temporal lobe epilepsy (TLE), thereby highlighting the considerable disparity in neuronal vulnerability to kainate (Nadler et al., 1978; Schwarcz et al., 1978b; Ben-Ari et al., 1980).

To investigate the occurrence and nature of “distant neurodegeneration” after a focal intra-striatal injection of kainic acid, Zaczek et al. (1980) used a silver impregnation technique to assess the integrity of neuronal cell bodies and axons distant from the injection site. Using [3H]-kainic acid as a tool, they observed a biphasic diffusion of the excitotoxin into adjacent structures with T1/2 values of 0.8 and 4 h. With the silver stain, they observed a finger of neuronal degeneration that extended into the pyriform cortex and the amygdala, affecting pyramidal neurons but sparing granule cells. Remarkably, neurons in the pyriform cortex and in the amygdala contralateral to the injection were often affected as well, indicating very high sensitivity. The role of seizures in this high, selective vulnerability of distant neuronal populations was supported by the demonstration that anticonvulsants provided neuroprotection (Zaczek et al., 1978).

Taken together, these studies confirmed the strategy of using *in situ* injections of the potent “glutamate receptor agonist” kainic acid to cause perikarya-specific and axon-sparing neuronal lesions in the adult brain. However, because of its pronounced pro-convulsive effects, and likely related to the fact that a significant proportion of kainate receptors is localized pre-synaptically on glutamatergic nerve terminals, kainate turned out not to be an optimal experimental tool in many cases. These properties accounted for its uneven neurotoxic effects, affecting neurons quite distant from the injection site, and for the age-dependence of kainate-induced excitotoxicity. These limitations undercut its utility as a universally suitable tool for producing *localized* excitotoxic lesions in experimental animals.

## The Ascendance of the Ibotenic Acid Lesion Method

In a study designed to examine and compare the excitotoxic properties of several neuroexcitatory amino acids known at the time, Schwarcz et al. (1978a) noted an important difference between kainic acid and ibotenic acid: although both showed quite similar excitotoxic potency, ibotenate did not induce behavioral seizures. In a follow-up study, Schwarcz et al. (1979) uncovered several other distinctions between ibotenate- and kainate-induced neurotoxity. While always producing “axon-sparing” lesions when injected *in situ* in adult rats, ibotenate, unlike kainate, not only failed to trigger behavioral convulsions but reliably caused uniform, spherical lesions of intrinsic neurons at the injection site in diverse brain regions including the striatum, hippocampus, substantia nigra and pyriform cortex. Notably, hippocampal granule and pyramidal neurons were found to be equally vulnerable to an *in situ* injection of ibotenate, and no damage to neurons in the pyriform cortex and the amygdala was observed in these animals; moreover, EEG studies raised the possibility that the weak epileptogenic properties of ibotenate may be the result of its metabolic conversion to the potent GABA_A_ receptor agonist muscimol (Aldinio et al., 1983). More generally, by being less toxic to the animals and by producing more discrete lesions, ibotenate soon became the preferred experimental agent for producing excitotoxic neurodegeneration in the brain.

The mechanism of action of ibotenate was further distinguished from kainate by the fact that ibotenate’s excitotoxicity was prevented by the selective NMDA receptor antagonists amino-phosphonovaleric acid and amino-phosphonoheptanoic acid whereas kainate was resistant (Schwarcz et al., 1982). This finding was not only of theoretical interest but led to the ground-breaking concept that selective inhibition of excitatory amino acid receptors could be used in the treatment of neurodegenerative and seizure disorders (Schwarcz et al., 1984; Schwarcz and Meldrum, 1985).

Curiously, and again in marked contrast to kainate, intra-striatal injection of ibotenate in the 7-day-old rat produced an extensive lesion of both intrinsic neurons and dopaminergic nerve terminals (Steiner et al., 1984). This inability to cause axon-sparing lesions during the early postnatal period is still poorly understood and limits the use of ibotenic acid as a lesioning tool in the developing brain.

## Kynurenines as Endogenous Modulators of Excitotoxicity

Stone and Perkins (1981), in exploring the neurophysiologic characteristics of glutamate analogs, found that quinolinic acid, a metabolite of the major route of tryptophan degradation (the “kynurenine pathway”), exhibited potent excitatory effects on cortical neurons. Marked sensitivity to amino-phosphonovaleric acid indicated that the NMDA receptor was the likely mediator of quinolinic acid-induced excitation. Schwarcz et al. (1983) then assessed the possible excitotoxic properties of quinolinic acid in the rat and demonstrated that intrastriatal injections of the metabolite indeed caused the degeneration of intrinsic GABAergic and cholinergic neurons without damaging afferent dopaminergic nerve terminals. Interestingly, the excitotoxic effects of quinolinic acid differed from both kainate and ibotenate. Like kainate, quinolinate was essentially non-toxic in the early postnatal period (Foster et al., 1983) and affected neurons differentially after focal injection in a variety of brain regions (Schwarcz and Köhler, 1983). On the other hand, quinolinic acid turned out to be only a relatively weak convulsant and therefore did not cause distant neuronal degeneration after *in situ* injection. These qualities were soon found to duplicate the neuropathological features of various human brain diseases with remarkable accuracy (Schwarcz et al., 1984; Beal et al., 1986), leading to the hypothesis that increases in the brain levels of *endogenous* quinolinic acid may play a critical, causative role in pathophysiology (Schwarcz et al., 1984).

The finding of Perkins and Stone (1982) that another metabolite of the kynurenine pathway, kynurenic acid, was a non-selective antagonist of excitatory amino acid receptors, was soon followed by the demonstration that this compound had neuroprotective properties, which were, interestingly, especially effective in inhibiting the excitotoxic effects of quinolinic acid (Foster et al., 1984). Following these early findings, one of us (RS) has spent the past decades defining the factors that affect the levels and function of endogenous quinolinic acid and kynurenic acid in the mammalian brain and exploring their potential roles in modulating excitotoxicity in a variety of human brain disorders (Schwarcz et al., 2012; Schwarcz, 2016). These studies, which also included additional neuroactive kynurenine pathway metabolites – but not the more prominent tryptophan metabolite serotonin – opened a whole new line of investigation of endogenous modulators of glutamatergic function and dysfunction. To this day, this work continues to reveal unforeseen new insights into brain physiology, with high relevance to the pathophysiology of a large number of diverse neurologic and psychiatric disorders (Kandanearatchi and Brew, 2012; Barone, 2019; Savitz, 2020).

## Conclusion (cf. Table 1)

The concept of excitotoxicity emerged over 60 years ago from the observation that systemic treatment with glutamate causes degeneration of neurons in the inner layers of the retina. Subsequent studies showed that glutamate-induced neuronal degeneration also occurred in a few discrete brain regions with deficient blood-brain barriers. Since the toxicity correlated with excitatory potency of glutamate analogs, Olney coined the term “excitotoxins.” To harness the lesion method so that it could be targeted at neurons intrinsic to any brain region, *in situ* injection of the most potent analogs of glutamate known at the time, kainic acid, was found to produce perikaryal-specific, axon-sparing lesion in the striatum that was mediated by a subtype of glutamate ionotropic receptors, the kainate receptor. However, kainate proved to be a potent convulsant resulting in highly variable neuronal vulnerability, which limited its use as a targeted excitotoxin. Ibotenic acid, which is also a potent excitatory agent but lacks the strong convulsant properties of kainate, proved ideal in causing perikaryal-specific, axon-sparing, spherical lesions regardless of brain region. The NMDA receptor-mediated mechanism of action of ibotenate led to the identification of the tryptophan metabolite quinolinic acid as an endogenous excitotoxin. Quinolinic acid and the metabolically related *anti-excitotoxic* (= neuroprotective) compound kynurenic acid are now regarded as major players in the pathophysiology of a wide range of neurological and psychiatric disorders.

## Author Contributions

Both authors wrote and edited the review.

## Conflict of Interest

The authors declare that the research was conducted in the absence of any commercial or financial relationships that could be construed as a potential conflict of interest.
